# Do the mutations of *C1GALT1C1 *gene play important roles in the genetic susceptibility to Chinese IgA nephropathy?

**DOI:** 10.1186/1471-2350-10-101

**Published:** 2009-09-24

**Authors:** Gui-Sen Li, Guang-Jun Nie, Hong Zhang, Ji-Cheng LV, Yan Shen, Hai-Yan Wang

**Affiliations:** 1 Renal Division, Department of Internal Medicine, Peking University First Hospital, and Peking University Institute of Nephrology, Beijing 100034, PR China; 2 Renal Division, Sichuan Medical Science Academy & Sichuan Provincial People's Hospital, Chengdu 610072, PR China; 3 Chinese National Human Genome Center, Beijing 100176, PR China

## Abstract

**Background:**

The deficiency of *β*1,3 galactose in hinge region of IgA1 molecule played a pivotal role in pathogenesis of IgA nephropathy (IgAN). Cosmc, encoded by *C1GALT1C1 *gene, was indispensable to *β*1,3 galactosylation of IgA1. We designed a serial study to investigate the relationship between the mutations of *C1GALT1C1 *gene and the genetic susceptibility to IgAN.

**Methods:**

Nine hundred and thirty-eight subjects, including 661 patients with IgAN and 277 healthy controls were enrolled in the study. Firstly, single nucleotide polymorphisms (SNPs) in the promoter region of *C1GALT1C1 *gene were screened. Then the c.-347-190G>A was analyzed by PCR-restriction fragment length polymorphism (PCR-RFLP) for further case-control association analysis. Secondly the somatic mutations of DNAs from peripheral blood B lymphocytes were detected in 15 patients and 7 normal controls.

**Results:**

No significant association was observed between the different alleles or genotypes of c.-347-190G>A and IgAN. The patients with different genotypes of *C1GALT1C1 *gene did not significantly associate with clinical manifestations, including hematuria, proteinuria, and serum creatinine of patients with IgAN. There was no somatic mutation detected in total 202 clones of 22 individuals.

**Conclusion:**

The c.-347-190G>A polymorphism and the somatic mutation of encoding region of *C1GALT1C1 *gene were not significantly related to the genetic susceptibility to IgAN in Northern Chinese population.

## Background

IgA nephropathy (IgAN), which is the most common glomerulonephritis and a leading cause for end-stage renal disease (ESRD) worldwide, is characterized by presence of IgA1 deposit in the glomerular mesangium. In recent years, aberrant glycosylations of IgA1 molecule in patients with IgAN were reported [1,2] and were considered as the most important pathogenic mechanism of IgAN [3-5]. Previous studies had demonstrated that circulating and glomerular deposited IgA1 in patients with IgAN showed deficiency of *β*1,3 galactose in the hinge glycopeptides [2,4,6]. The deficiency of hinge-region glycosylation of serum IgA1 showed an increased tendency to self-aggregation and/or the increased binding to circulating glycoproteins and enhanced reaction with specific IgG antibodies directed against IgA1 hinge O-glycans. These IgA aggregates could escape the clearance by hepatic receptors for asialoglycoproteins [4]. Others' and our previous studies demonstrated that IgA1 with deficiency of hinge-region *β*1,3 galactose had a higher binding capacity and stronger biologic effects to cultured human mesangial cells, leading to accumulation and/or prolonged deposit of IgA within the mesangium [7-9]. The *β*1,3 galactose deficiency of serum IgA1 were closely associated with renal pathologic phenotypes of IgAN [10]. Therefore, deficiency of hinge-region *β*1,3 galactosylation of IgA1 molecule might play a pivotal role in the pathogenesis of IgAN.

The mechanisms contributed to aberrant galactosylation of IgA1 molecule in patients with IgAN were still unclear. In fact, the core 1 structure Gal*β*1→3GalNAcα1- Ser/Thr (T antigen) was synthesized from GalNAcα1-R (Tn antigen) by the action of core 1 *β*3-galactosyltransferase (C1*β*3Gal-T). The coding gene of C1*β*3Gal-T was *C1GALT1*. However, there was no apparent disparity of *C1GALT1 *expression among normal controls, non-IgAN glomerulonephritis, and IgA nephropathy [11]. Further studies revealed that the C1*β*3Gal-T activity required expression of a molecular chaperone designated Cosmc (core 1*β*3-Gal-T-specific molecular chaperone) [12]. *C1GALT1C1 *(OMIM*300611), the coding gene for Cosmc [12,13], was mapped to chromosome Xq23, included 3 exons and spanned about 4 kb [12,13]. Mutations of *C1GALT1C1 *could impressively changed the enzyme activity of C1*β*3Gal-T [12,14]. But our previous study in patients with IgAN revealed that there was only one mutation (c.393T>A) detected in the coding region of the *C1GALT1C1 *gene [15]. The minor allele frequency (MAF) of c.393T>A was only 6.90% [15]. In additionally, a previous study had revealed that the conservative amino-acid substitution (Asp131→Glu) which derived from the c.393T>A mutation gave normal C1*β*3Gal-T activity [14]. However, other two somatic mutations of *C1GALT1C1 *gene contributed to the aberrant galactosylation of Tn syndrome [14]. Malycha et al [16] found that none of *C1GALT1C1 *mutations played important roles in the pathogenesis of IgAN in a rencent study. It was unclear whether there were any losses of function mutations or somatic mutations of *C1GALT1C1 *gene in Chinese IgAN patients.

We hypothesized that the mutations of *C1GALT1C1 *gene could influence its activities in two pathways: mutations in the promoter region or somatic mutations in the coding region. To prove the validity of the hypothesis, firstly, we screened the mutations in the *C1GALT1C1 *gene in the promoter region and used a case-control association analysis to test the relationship of the polymorphisms and the susceptibility or clinical manifestations of IgAN. Secondly, somatic mutations of *C1GALT1C1 *gene were detected in patients with IgAN.

## Methods

### Subjects

A total of unrelated 938 northern Chinese were involved in this study, including 661 patients with IgAN proved by renal biopsy, and 277 geography-matched healthy controls with normal urine analysis and blood pressure. Patients with Henoch-schonlein purpura, systemic lupus erythematosus, and chronic hepatic diseases were excluded by detailed clinical and laboratory examinations. The mean ages were 31.2 ± 11.4 (patients) and 28.9 ± 8.2 (the controls) years. There were 82 female subjects in the control group and 280 female subjects in the IgAN patient group. Twenty-two individuals (15 patients who were recently diagnosed as primary IgAN and 7 controls) were selected for somatic mutation detection.

Clinical data of patients with IgAN, including age, course of kidney disease before renal biopsy, as well as blood pressure and the level of urine protein excretion at the time of renal biopsy, were collected. At the same time, the renal function was evaluated, including serum creatinine and estimated glomerular filtration rate (eGFR) calculated by the Modification of Diet in Renal Disease abbreviated equation [17].

The protocol for this study was approved by medical ethics committee of Peking University, and informed written consents for the study were obtained from all participants.

### SNPs Discovery and Genotyping

Genomic DNA of subjects was extracted from the EDTA-anticoagulated whole blood samples by salting out procedure [18]. The *C1GALT1C1 *gene was located in the chromosome X. Reference sequence of *C1GALT1C1 *gene (OMIM*300611, Version: NC_000023.9) was obtained from National Center for Biotechnology Information (NCBI) Gene database . Fifty-eight alleles from chromosome X were screened in all of the 46 individuals, including 27 unrelated patients with IgAN (6 females) and 19 unrelated healthy controls (6 females). The polymerase chain reaction (PCR) amplification regions included 5' untranslated regions and the upstream 1 kb from transcriptional initiation site. PCR primers were designed by Primer3 program [19]. Target sequences were amplified by PCR from 50 ng genomic DNA in 20 μl of final reaction volume. DNA sequencing was performed on an ABI PRISM 3700 automated sequencer. The results of sequencing were analyzed by Phred/Phrap/Consed suite of software [20]. The SNPs were described according to the Human Genome Variation Society (HGVS) nomenclature guidelines [21].

One single nucleotide polymorphism (SNP), c.-347-190G>A (rs3810744), was detected in the promoter region of the *C1GALT1C1 *gene. The MAF of c.-347-190G>A (rs3810744) was 48.48%. So the SNP was genotyped for further association analysis in all 938 subjects by the standard PCR-restriction fragment length polymorphism procedures. The genomic DNA samples were amplified by PCR using the following primers, forward: 5'-ACGCAGGGGTACATCAGAGAA-3', reverse: 5'-TGACCAGGCTGTTCTAGCTG-3'. The products of 420 base pairs (bps) were digested by restriction endonuclease Hpy8I (Fermentas International Inc., Hanover, USA). The genotypes weren't detected in three controls and one patient. Forty PCR products were sequenced for accuracy confirmation of PCR-restriction fragment length polymorphism analysis.

### B lymphocyte DNA extraction and PCR amplification

Peripheral blood B lymphocytes from 22 participants (15 patients and 7 controls) were isolated by using lymphocyte isolation sterile solution (Amersham Biosciences, Uppsala, Sweden) and CD19 magnetic beads (Dynal Biotech ASA, OSLO, Norway), and then DNA of B lymphocytes was extracted by salting out procedure [18]. The whole coding region was amplified by PCR with following primers, forward: 5'-GTTGTTGCAAAGTTCCAGTTTA-3', reverse: 5'-TTATACCAGTGCCACCAAATTA-3'. The length of PCR production was 1314 bps. B lymphocyte DNA (50 ng) was amplified in a final reaction volume of 50 μl, containing 10 pmol of each primer and 2U Pyrobest DNA polymerase (Takara Biotechnology, Japan). Touchdown PCR procedure was used as followings: the initial denaturation at 95°C was followed by 30 cycles of denaturation at 95°C for 30 sec, annealing at 68°Cfor 35 sec (the temperature decrease 0.5°C each circle), and elongation at 72°C for 100 sec, then 18 cycles of denaturation at 95°C for 30 sec, annealing at 53°C for 35 sec, elongation at 72°C for 100 sec, finally elongation at 72°C for 7 min.

### Gene cloning and somatic mutation detection

PCR products from B lymphocyte DNA of 22 individuals were subcloned into PGEM-T vector (Promega Corporation, Madison, WI, USA) after purification and adding adenine to them. Then ligation productions were transformed to Ecoli Top 10 competent cells and cultured in Luria-Bertani (LB) solid medium for 14 hours at 37°C. More than 8 clones per individual were randomly selected and amplified in LB liquid medium for 14 hours at 37°C. Plasmids were extracted and digested with PST1 restriction enzyme (Promega Corporation, Madison, WI, USA) to verify the insertion of PCR productions. Total 202 clones, including 8 to 10 clones per individual, were directly sequenced to detect somatic mutation.

### Statistical Analysis

Observed genotype frequencies in female subjects for all case and control groups were tested for Hardy-Weinberg equilibrium using χ2 tests with 1 df. Data were expressed as percentages or mean ± standard deviation. Pearson's χ2 was used for categorical data. Continuous variables were tested in each group for normal distribution using the Kolmogorov-Smirnov test for one variable. Differences of the means between two groups were tested with Student's t test. The means among the three groups were compared by ANOVA analysis. Statistical analysis was performed by SPSS 10.0 program (SPSS Inc., USA). All tests were two-sided and a *P *value of less than 0.05 was considered statistically significant.

## Results

### Detection of polymorphisms and association study

One SNP, c.-347-190G>A (rs3810744) was detected in the promoter region. And then an association analysis was performed in the cases and controls for the SNP. The frequencies of alleles and genotypes were presented in table 1. The difference in allele frequencies between male and female controls were not significant (A allele: 0.467 vs. 0.500, *P *= 0.533). No significant associations were observed between alleles and IgAN, whether in total (*P *= 0.121), in male (*P *= 0.684), or in female samples (P = 0.085). The association between genotypes of c.-347-190G>A and patients with IgAN was significant only in female population. The frequency of GG/GA genotype was higher in patients than in controls (0.781 vs. 0.645, *P *= 0.033).

**Table 1:** Distributions of c.-347-190G>A Polymorphism in *C1GALT1C1 *Gene

	**All**		**Males**		**Females**				
	
	**G**	**A**	**G**	**A**	**GG**	**GA**	**AA**	**G**	**A**
Controls	183	170	104	91	28	23	28	79	79
Cases	532	407	210	171	104	114	61	322	236
*P *value	0.121		0.684		0.033			0.085	

### C1GALT1C1 gene c.-347-190G>A polymorphism and clinical manifestations or prognosis in patients with IgAN

Clinical characteristics of patients with IgAN at the time of renal biopsy were listed in Table 2. The patients were divided into two groups according to their genders. When clinical parameters of two male groups with different genotypes were compared, there was not significant difference in age, course of disease, incidence of gross hematuria or high blood pressure, urine protein excretion in 24 hours, serum creatinine concentration, and serum levels of IgA. These clinical parameters also did not differ significantly among the three female groups with different genotypes.

**Table 2:** General clinical parameters of IgAN patients with different c.-347-190G>A genotypes of *C1GALT1C1 *gene

	**Males**		**Females**		
	
	**G**	**A**	**GG**	**GA**	**AA**
	
Age, years	29.6 ± 11.7	30.8 ± 11.7	33.0 ± 10.7	32.6 ± 11.5	31.8 ± 9.9
Courses of diseases, months*	5.0(16.2)	4.0(14.7)	6.0(22.0)	6.0(22.3)	6.0(15.0)
Incidence of gross hematuria, %	27.1	27.8	44.3	44.9	35.1
Incidence of hypertension, %	51.2	48.8	25.0	36.4	33.3
Systolic blood pressure, mmHg	129 ± 20	128 ± 20	117 ± 19	122 ± 23	118 ± 19
Diastolic blood pressure, mmHg	82 ± 15	81 ± 14	74 ± 14	78 ± 14	76 ± 14
Urinary protein excretion, g/day	2.76 ± 3.44	2.14 ± 2.18	2.19 ± 3.26	2.00 ± 2.64	2.11 ± 2.84
Serum creatinine, μmol/l	123.3 ± 95.1	140.6 ± 170.6	89.4 ± 89.9	83.5 ± 40.1	86.0 ± 81.0
eGFR^#^, ml/min/1.73 m^2^	67.7 ± 35.0	62.3 ± 29.9	87.1 ± 31.6	86.3 ± 30.3	90.7 ± 35.2
Serum albumin, g/l	37.8 ± 6.9	39.0 ± 7.0	37.4 ± 6.2	38.2 ± 5.3	35.8 ± 7.7
Serum IgA, g/l	2.94 ± 1.27	3.02 ± 1.04	3.00 ± 1.06	3.06 ± 1.35	2.93 ± 1.23

All the parameters were detected at the time of renal biopsy; ^# ^eGFR, estimated glomerular filtration rate; * Median (Q_R_).

### Somatic mutation detection of B lymphocyte DNA

Although more than 8 clones per individual were sequenced for the whole coding region, neither new mutation nor new polymorphism except c.393T>A (rs17261572) was detected in total 202 clones from B lymphocyte DNA in 22 individuals (15 patients and 7 controls). The A allele of c.393T>A was only detected in each clone in two patients with IgAN and one male control. One of the two patients was male and another one was a female with AA homozygote. The results were completely consistent with the sequencing results derived from the previous genomic DNA.

## Discussion

IgA nephropathy, the most common primary glomerulonephritis, was considered as a polygenic and multifactorial disorder. There were extensive evidences suggested the genetic components were involved in the susceptibility and progression of IgAN [5,22-24]. The pathogenesis of IgAN was still indistinct, as far as we knew. Fortunately, more and more evidences suggested that deficient *β*1,3 galactosylation of hinge region of IgA1 molecule played an important role in the pathogenesis of IgAN in recent years [4,5].

The galactosylation of GalNAcα1-R in hinge region of IgA1 molecule depended on the activity of C1*β*3Gal-T. Intriguingly, patients with IgAN had normal expression of *C1GALT1 *gene and decreased expression of *C1GALT1C1 *gene [11]. Furthermore, diseases resulted from deficiency of *β*1,3 galactose, such as Tn syndrome, weren't a result of decreased expression of *C1GALT1 *gene, but resulted from the mutations of *C1GALT1C1 *gene[14]. These results suggested that it was rather the variants of *C1GALT1C1 *gene in influencing the galactosylation of IgA1 hinge-region than the variants of *C1GALT1 *gene. It implies that the variants of *C1GALT1C1 *gene could contribute to susceptibility of IgAN by influencing *β*1,3 galactosylation of IgA1 molecule. Our previous study revealed that there was only one SNP, c.393T>A (rs17261572), in coding region of *C1GALT1C1 *[15]. It was a nonsynonymous SNP. The MAF of c.393T>A was only 0.069. A previous study revealed that the mutations weren't important for the European IgAN patients [16]. Were the mutations (including somatic mutation) in the promoter region important in the pathogenesis of IgAN in China? Therefore, we designed a study to test the hypothesis.

We firstly screened the polymorphisms of *C1GALT1C1 *gene in promoter region. One SNP, c.-347-190G>A was detected. And its MAF was 48.48%. Therefore, association between the c.-347-190G>A polymorphism and IgAN was explored in a case-control association study in a large population sampled from the Northern Chinese. The association analysis revealed that there was no significant difference of the alleles between the controls and the IgAN patients in total samples or in two sub-group samples divided by genders. There was only a weak association between the genotypes of c.-347-190G>A (GG/GA) and IgAN detected in female cases. But the positive association wasn't replicated in male sample simultaneously. The *C1GALT1C1 *gene located on chromosome X, so the effect of polymorphisms would be influenced by the inactivity of sex chromosome. The positive association might not demonstrate a truly causal association between the SNP and IgAN. These results suggested that polymorphisms of *C1GALT1C1 *gene might not be related to the susceptibility of IgAN.

In present study, we further analyzed the association between the SNP of *C1GALT1C1 *gene and clinical parameters of IgAN. The results revealed that there was no significant difference of blood pressure, proteinuria, and renal function among the IgAN patients with different genotypes. These data suggested that the genotypes of the *C1GALT1C1 *gene did not influence the clinical manifestations of IgAN.

In previous studies of Tn syndrome, three somatic mutations of *C1GALT1C1 *gene were identified in two patients. The three somatic mutations, c.202C>T, c.393T>A and c.454G>A, were all in the coding region of *C1GALT1C1 *[14]. Except the c.393T>A mutation, both of the other two somatic mutations could impressively inhibit chaperone activity and lead to inactivation of C1*β*3Gal-T, and the expression of autoimmune Tn antigen on blood cells of all lineages[14]. Galactosylation deficiency was already proved in patients with IgAN. Does somatic mutation exist in *C1GALT1C1 *gene in patient with IgAN too?

In order to prove this hypothesis, we furthermore performed a somatic mutation screening in the patients with IgAN. DNAs from B lymphocytes where IgA molecule was produced were isolated from 22 individuals. And then the coding region of *C1GALT1C1 *gene was amplified, cloned and sequenced. Except the c.393T>A, no other mutations were detected. The mutation, c.393T>A, was only found in the patients whose mutations were demonstrated in genomic DNA by routinely sequencing. Furthermore, c.393T>A was proved not to be a somatic mutation in these IgAN patients. The result indicated that the variation of coding region of *C1GALT1C1 *gene might be of little importance in the processing of aberrant glycosylation of IgA1 molecule in patients with IgAN. Mutations in other regions of *C1GALT1C1 *gene, which may influence the glycosylation process of IgA1 molecule, were needed to be clarified in patients with IgAN. In fact, in a recent study, Malychaet al [16] detected mutations in whole blood DNA and in B cell DNA separately in a relative small European sample. They didn't found any important mutations of *C1GALT1C1 *gene in patients with IgAN. These results suggested that the *C1GALT1C1 *gene might influence the susceptibility to IgAN by an alternative pathway if it was important for IgAN.

## Conclusion

Our results suggested that the mutation (including somatic mutation) of *C1GALT1C1 *gene did not significantly contribute to the genetic susceptibility or clinical manifestations of IgA nephropathy in Chinese population.

## Competing interests

The authors declare that they have no competing interests.

## Authors' contributions

Both GSL and GJN carried out the molecular genetic studies and GSL performed the statistical analysis and drafted the manuscript. GSL, HZ, YS and HYW participated in its design. HZ, YS and HYW participated in its coordination. JCL and HZ participated in the acquisition of data and follow-up. HZ and HYW revised the manuscript critically for important intellectual content. All authors read and approved the final manuscript.

## Pre-publication history

The pre-publication history for this paper can be accessed here:


